# Multiple field tests on landing day: Early mobility may improve postural recovery following spaceflight

**DOI:** 10.3389/fphys.2022.921368

**Published:** 2022-09-14

**Authors:** Marissa J. Rosenberg, Millard F. Reschke, Elena S. Tomilovskaya, Scott J. Wood

**Affiliations:** ^1^ KBR, Houston, TX, United States; ^2^ Neurosciences Laboratory, NASA Johnson Space Center, Houston, TX, United States; ^3^ Institute of Biomedical Problems of the Russian Academy of Sciences, Moscow, Russia

**Keywords:** vestibular, rehabilitation, posturography, sensorimotor, incremental

## Abstract

Adaptation to microgravity causes astronauts to experience sensorimotor disturbances during return to Earth leading to functional difficulties. Recently, the Field Test (FT) study involving an incrementally demanding sensorimotor functional test battery has allowed for an unprecedented view into early decrements and recovery from multiple tests conducted on the landing day following 6-months International Space Station missions. Although the protocol was challenging and temporarily increased motion sickness symptoms, there were anecdotal reports that performing these tasks within the first few hours of landing accelerated their recovery. Therefore, results from computerized dynamic posturography (CDP) following return to Houston were used to compare recovery between crewmembers that participated in FT (*n* = 18) with those that did not (controls, *n* = 11). While there were significant decrements in postural performance for both groups, some FT participants tended to perform closer to their preflight baseline in the most challenging condition of the CDP sensitive to vestibular function—eyes closed, unstable support and head movements. However, the distribution of difference scores appeared bimodal with other FT participants in the lower range of performance. We attribute these observations to the manner in which the field tests were implemented—some benefitted by encouraging early movement to drive adaptation when performed in a constrained incremental fashion; however, movements above aversive thresholds may have impaired adaptation in others. Challenging the sensorimotor system with increasingly provocative movements performed as close to landing as possible, as long as within individual thresholds, could be a useful intervention to accelerate astronaut’s sensorimotor readaptation that deserves further study.

## 1 Introduction

Alterations in sensorimotor processing during spaceflight lead to performance decrements in functional tasks following transitions from microgravity to a gravitational environment. The greatest decrements in performance occur during functional tasks that require dynamic control of postural equilibrium (Miller et al., 2018; Mulavara et al., 2018). Exercise countermeasures available on the International Space Station (ISS) especially those conducted late inflight appear to improve recovery (Kozlovskaya et al., 2015; Loehr et al., 2015); however, competing constraints on exploration vehicles will limit future inflight countermeasures available (Chavers et al., 2021). Therefore, countermeasure strategies are needed to enhance sensorimotor adaptation to mitigate risks following landing on planetary surfaces where external support will not be available.

We propose that early mobility with incrementally increasing sensorimotor challenges, as long as movements are kept within one’s motion tolerance, may optimize adaptation to the new gravitoinertial environment. This is based in part on evidence from cerebellar neurons that comparison of actual and predicted sensory feedback during voluntary self-motion appears to be critical in updating internal models associated with motor learning (e.g., Brooks et al., 2015). In particular, motor learning tasks that incorporate incremental error signals are more effective in driving neural plasticity and learning (Kagerer et al., 1997; Cakit et al., 2007; Schubert and Migliaccio, 2019). In addition to an incremental approach involving active movements, an early intervention following the G-transition may be equally important. Vestibular rehabilitation following acute peripheral loss appears to benefit from earlier exercises (Michel et al., 2020) in the same way that earlier mobility can improve rehabilitation outcomes in intensive and intermediate care settings (Drolet et al., 2013; Dirkes and Kozlowski, 2019).

Exercises with increasing levels of difficulty customized to an individual’s state of recovery is consistent with our post-landing strategy (Wood et al., 2011). However, the supervised reconditioning program is typically delayed by more than 1 day while crewmembers return from the Soyuz landing site in Kazakhstan. Field Tests (FT) were conducted at the landing site to quantify functional postflight performance following long duration missions lasting ∼6 months and track their recovery (Reschke et al., 2020). While not designed to be a rehabilitation-type study, the testing constraints followed similar guidelines as we propose. Participants performed a series of incrementally more difficult mobility-related tasks at both the landing site and the refueling stop during their direct return. Tasks were not completed when the motion would be considered above an aversive threshold (e.g., elicit vomiting). The intent to capture initial decrements as close to landing as possible ensured an earlier implementation of the protocol.

The purpose of this paper was to determine if these early, multiple testingon landing day improved postural recovery in the participating crewmembers compared to those who did not participate. Specifically, we compared measures between groups using Computerized Dynamic Posturography (CDP) measures conducted the day after landing (Wood et al., 2015). Based on the most challenging CDP test conditions requiring effective use of vestibular input (standing eyes closed on unstable surface with head erect or performing pitch head tilts), postflight postural recovery appeared improved in some field test participants versus non-participant controls. However, the bimodal nature of responses suggest that others may have pushed beyond their motion tolerance limit in an effort to complete more FT objectives. These observations are consistent with encouraging early movement to drive adaptation but performed in a constrained fashion to minimize movements above aversive thresholds.

## 2 Methods

### 2.1 Subjects and timeline

Twenty-nine United States Orbital Segment (USOS) astronauts returning from long-duration ISS missions in the 2010–2020 timeframe were included in our analysis. Eighteen subjects participated in the FT protocol described below, and data from 11 control astronauts who did not perform FT were obtained from NASA’s medical data repository. FT participants include nine in a pilot FT (PFT) protocol and nine in the full protocol (full FT). Two of the control subjects overlap groups, with one participating in the PFT on an earlier mission and the other as a full FT participant in a later mission. For both of these crewmembers, their flights were several years apart. Although we expect some dependence between the same individual on different missions, these were included to maximize the subject pool per cohort. Specific expedition numbers or lengths of missions were not referenced to minimize the risk of data attributability according to NASA policy. While FT were conducted on both USOS astronauts and Russian cosmonauts (Reschke et al., 2020), we limited this analysis to USOS subjects who had multiple tests on landing day and early CDP data were available. The control group had similar male/female ratio, age range, flight experience, mission duration and timing for the postflight CDP test as the FT groups (Table 1).

**TABLE 1:** Demographics of the three cohorts, those who participated in Pilot Field Test, full Field Test, and Controls. Note that PFT and Full FT cohorts have been combined for final analyses.

Cohort	Subject count (male/Female)	Age (y, mean ± std)	Flight number (mean, range)	Mission duration (d, mean ± std)	Time of CDP tests (h, mean ± std)
Pilot FT	9 (8M, 1F)	46.7 ± 6.1	2.2, 1–4	179 ± 15	40.9 ± 5.8
Full FT	9 (8M, 1F)	50.2 ± 5.8	1.9, 1–4	167 ± 25	31.8 ± 9.0
Control	11 (10M, 1F)	48.2 ± 3.3	2.0, 1–3	159 ± 23	34.8 ± 4.3

Crewmembers were typically assisted out of the capsule and carried to the medical tents at the Soyuz landing site for assessments and field testing, and then assisted to helicopters to be flown to the nearby rally airport. Nominally, all subjects completed PFT or FT at the Soyuz landing site (Kazakh Steppe) in the medical tent within 1–2 h of landing. If the medical tent was not deployed at the landing site or tests could not be performed there, tests were performed at an airport 4–5 h after landing. This test session occurred an average of 2.32 ± 1.3 h after landing. All USOS crewmembers were then assisted to the Gulfstream aircraft to be flown from Kazakhstan to Houston, TX. The aircraft is equipped with couches converted into beds to allow sleep and medical supplies to provide intravenous fluid therapy and medications as needed (Patlach and Alexander, 2013; Lee et al., 2020). One refueling stop in western Europe provided the opportunity for a second test session en route, on average 13.4 ± 0.8 h after landing. Both FT and no-FT participants typically ambulated with assistance during the refueling stop. Following the return flights, there was ambulation at Ellington Field and the astronaut crew quarters before transport to the testing facilities at the Johnson Space Center (JSC). The CDP session was then performed at JSC between 23–51 h (35.8 ± 6.9 h, mean ± std) after landing, with variations due to flight and testing constraints. All subjects gave informed consent according to the requirements of the Institutional Review Boards.

### 2.2 Field test

The specific Field Test protocol has been described elsewhere (Lee et al., 2020; Reschke et al., 2020). The common mobility tasks performed across both PFT and full FT protocols included sit-to-stand, recovery from fall (prone to stand) and tandem walk, performed in that order of increasing difficulty. For the sit-to-stand, crewmembers stood up without using their hands and remained standing for 10 s. The recovery from fall involved rising from a prone position and standing for up to 4 min. The tandem walk was the most challenging and performed last, requiring 10 heel-to-toe steps with arms crossed, and repeated with eyes closed and open. Full FT also included a timed up and go mobility test (sit-to-stand, walk 4 m, turn 180° and return to seated) with small obstacles (5–15 cm height) to step over on the return path (Reschke et al., 2020). Additional full FT tasks included a standing posture test with an upper body perturbation (push) and passive dynamic visual acuity during vertical linear oscillations on a spring-loaded chair. There were also a variety of seated tasks (eccentric gaze, dysmetria finger to nose, eye-hand coordination on a tablet, grip force discrimination) that were interspersed in the full FT testing protocol. If crewmembers were not comfortable performing the more difficult functional tasks, they were allowed to perform the seated tasks alone. Four of the 18 FT participants included in this analysis were not able to complete the full test battery due to motion sickness at the landing site and refueling airport. As stated above, stopping activity outside of one’s motion threshold is a key feature of the incremental rehabilitation approach we are recommending.

### 2.3 Computerized dynamic posturography

CDP measures were conducted as part of medical assessments used to quantify the initial postflight decrements and recovery of postural stability (Wood et al., 2015). Multiple preflight CDP tests were conducted to minimize the effects of learning, and the preflight measurements used in this analysis were obtained from the last preflight session, usually 3 months before launch. CDP was conducted using a modified EquiTest system (NeuroCom International, Clackamas, OR). Subjects were instructed to maintain stable upright posture with arms folded across the chest. This early postflight session is limited to two Sensory Organization Test conditions with eyes closed, sway-referenced base of support, with three trials of head erect (SOT-5) followed by three trials of head moving (SOT-5M). The sway-referenced rotations of the support surface about the ankle joint are directly proportional to anterior-posterior (AP) sway to disrupt proprioceptive feedback. With this unstable platform and eyes closed, these conditions are the most sensitive to disruptions in vestibular processing and have the greatest diagnostic accuracy in detecting postflight decrements (Jain et al., 2010). Subjects wore noise-cancelling headphones through which operator instructions and white noise were supplied to mask external auditory orientation cues. Sinusoidal pitch head movements were paced at 0.33 Hz by an audible tone at ±20° guided by operator using feedback from a motion tracker mounted to the headphones (MTx, Xsens Technologies, Netherlands).

The AP peak-to-peak sway angle was used to compute a continuous equilibrium (cEQ) score between 0 and 100 that factors in the time before a fall occurs, thus separating ballistic falls from falls that occurred later in the trial (Wood et al., 2012). Falls were marked when subjects moved their feet, began to take a step, or raised their arms. The median cEQ score of the three trials were calculated for both SOT conditions, and the delta cEQ scores were computed (Post-Pre) with higher numbers representing better performance. Goodness of fit to normal distributions were evaluated with Shipiro-Wilk statistic. Due to the skewed nature of the cEQ scores, non-parametric Wilcoxon signed-rank test were used for comparing paired pre-to-postflight differences, the Mann-Whitney test for comparing FT and control independent groups, and Spearman Rank correlation (*r*_*s*_) for examining strength of relationships. Based on the Mann-Whitney test statistic, we calculate the probability of superiority (PS), or the probability of an observation in the FT group having a true value that is higher than an observation in the non-FT group, as a measure of effect size (Conroy, 2012).

## 3 Results

**FIGURE 1:**
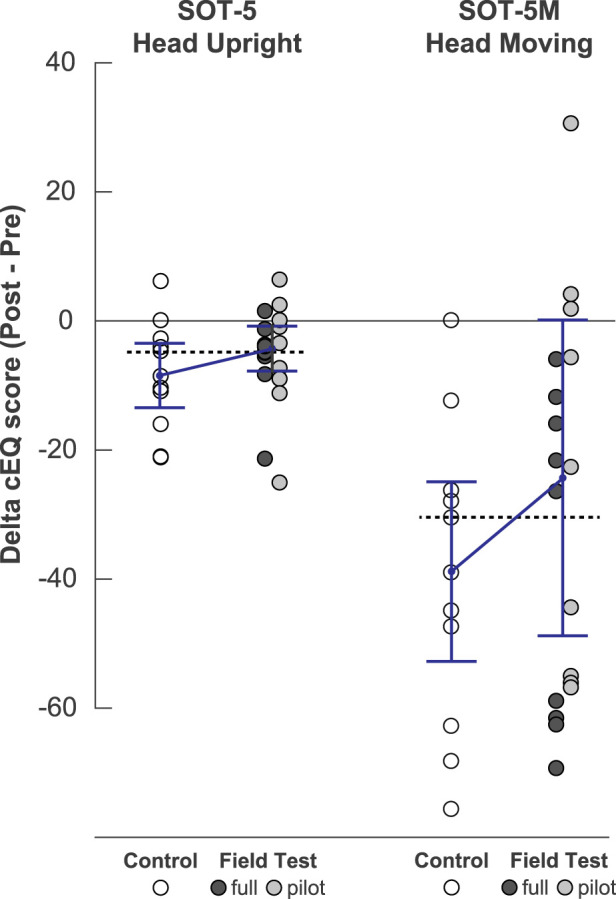
Comparison of postflight postural performance in FT participants and controls. The left panel is the SOT 5 condition (eyes closed, sway-reference support, with head upright). The right panel is the SOT-5M condition (eyes closed, sway-referenced support, with pitch head movements). Individual data points represent the difference between pre- and post-flight Equilibrium scores, with medians ± IQR for each group overlaid. For reference, the dashed line represents the grand median for all groups.

Unless otherwise stated, the PFT and full-FT cohorts have been combined for this analysis. The distribution of cEQ scores with FT and control groups often deviated from normality based on Shapiro-Wilk statistic (*p* < 0.05 criteria) reflecting the skewed nature of the cEQ measures. While the delta cEQ scores passed this normality criteria, postflight scores did appear slightly bimodal (Figure 1) reflecting the variability in responses. Preflight performance was similar across groups. For SOT 5, preflight median cEQ score (±IQR) was 85.6 ± 6.5 for control and 90.6 ± 4.7 for FT (*p* = 0.91). For SOT 5M, preflight median was 78.6 ± 6.3 points for control and 81.8 ± 19.6 for FT participants (*p* = 0.51). Postflight two FT subjects completed only 2 of 3 SOT-5M trials; otherwise, all participants completed three trials for both SOT5 and SOT-5M in all sessions. Based on paired Wilcoxon signed-rank tests, FT and control groups had significant pre-to-postflight decrements in cEQ for both SOT5 (*p* < 0.01) and SOT5M (*p* < 0.001).

While these group decrements are consistent with previous ISS findings (Wood et al., 2015), from inspection of Figure 1 it is evident that some FT participants tended to perform closer to or even better than their preflight baselines. The median (±IQR) delta cEQ scores for SOT-5 for the controls were -8.5 ± 10.1 compared to -4.4 ± 7.0 for the FT group (z = 0.99, *p* = 0.32). The mean delta cEQ scores for SOT-5M for the controls were -39.0 ± 28.0 -compared to −24.5 ± 49.2 for the FT group (z = 1.03, *p* = 0.30). The probability that an observation from the FT group (with both full and pilot subgroups combined) was greater than the control group was PS = 0.61 for SOT-5 and PS = 0.62 for SOT-5M. Note that the two participants included in both FT and control groups performed better following FT participation. Improvements in post-flight performance were noted among both PFT and full-FT cohorts, although the greatest difference was between the pilot-FT and controls during SOT-5M (z = 1.25, *p* = 0.21, PS = 0.67). Nevertheless, the distribution of difference scores in the FT group, particularly for SOT-5M, appeared bimodal with some FT participants among the most impaired (Figure 1). The observation that some of the worst FT performances on SOT-5M were in the full-FT subgroup may reflect that the additional tasks required for the full protocol were more likely to exceed motion thresholds for some participants. Among other factors that may have contributed to postflight performance, we found that the number of flights (*r*_*s*_ = −0.02, *p* = 0.92), mission duration (*r*_*s*_ = 0.01, *p* = 0.95) and timing of the post-flight CDP (*r*_*s*_ = 0.02, *p* = 0.93) were all not correlated with the delta cEQ scores for SOT-5M across FT and control cohorts.

## 4 Discussion

Our post-flight CDP measures reflect the high intersubject variability that characterize postural decrements following spaceflight (Wood et al., 2015). This variability is consistent with other measures obtained during the Field Tests (Reschke et al., 2020) as well as previous studies (Miller et al., 2018; Mulavara et al., 2018). Nevertheless, multiple test sessions on landing day, starting early at the recovery zone, anecdotally appeared to be beneficial for some participants. Comparison of FT and no-FT participants in the most vestibularly challenging CDP condition support these anecdotal reports. We infer from these observations that performing minimal, challenging sensorimotor tasks very early in recovery provided enough challenge to the sensorimotor system to accelerate readaptation in some crewmembers.

This incidental discovery is not particularly surprising. Early ambulation is known to improve recovery outcomes following surgical interventions (e.g., Oldmeadow et al., 2006). While an early intervention is complicated by increased motion sensitivity at landing, similar interventions have proven useful clinically with motion sensitive vestibular patients (e.g., acute peripheral loss, Michel et al., 2020). This is also consistent with the observation that systematically increasing head movements during Shuttle reentry, as long as maintained within one’s threshold for motion tolerance, anecdotally appeared to improve recovery (Wood et al., 2011). Performing head tilts too rapidly or with too much amplitude can exacerbate symptoms and illusory sensations (Small et al., 2012); conversely, restricting head movements can delay readaptation. The seemingly bimodal distribution of responses suggests that FT participation did not improve recovery in all subjects, illustrating the importance of maintaining activity within an individual’s threshold. Our test protocol generally followed an incrementally challenging test sequence. Further improvements would be expected if the focus were on rehabilitation and customization of task difficulty.

There are limitations of this type of retrospective analysis. First, there is the possibility of self-selection bias for those consenting to participate in the Field Tests. Since ambulation was not quantified apart from the test sessions, it is unknown how much difference there was between groups. As noted in the methods, there was assisted ambulation for all participants who stood from sitting and lying positions, showered, and used stairs at the airports as part of their daily activities. Participating in the Field Tests likely had the greatest impact in early ambulation at the medical tents. Our comparison is made within an operational context with different medical interventions across subjects (Lee et al., 2020). The limited sample available as well as variations in CDP postflight test schedule also limit group comparisons.

The potential benefits from early mobility on landing day can be inferred from both anecdotal reports of the participants and comparison of the postural performance with no-FT participants. This finding has implications for exploration design reference mission planning. Instead of delaying planetary surface operations to allow for recovery, our results suggest that early mobility may be important. Rehabilitation should be optimized, as the tasks performed during Field Test were created to simulate aspects of mission-critical functional movements and were not intended as rehabilitation. Early active retraining, individualized based on the level of initial impairment, will enable a more efficient motor learning to the new environment (Lacour, 2006). Additionally, the rehabilitation should be phased appropriately, starting with simple tasks that grow in complexity with ability and time. A self-administered approach should provide optimized, graded tasks, e.g., beginning with low range-of-motion movements such as finger-to-object targeting practice and small postural changes, and advance to dynamic balance challenges. These should be performed as soon to landing as possible. It is also critical that the astronaut is coached to never exceed a motion sickness level around malaise, as once surpassed nausea and vomiting may not subside for hours. The importance of structuring rehabilitation exercises and early operational activities using *individualized* aversive threshold limits is underscored by our participants who may have impaired their recovery in an effort to complete all FT tasks, even when some tasks provoked motion sickness. These guidelines derived from our Field Test experience provide a framework to optimize performance for early mission success following G-state transitions during future space exploration. The bimodal response to FT participation has implications for vestibular rehabilitation on Earth; namely that early retraining must be individualized to promote adaptation while avoiding aversive conditioning.

## Data Availability

The datasets presented in this study can be found in online repositories. The names of the repository/repositories and accession number(s) can be found below: NASA Life Science Data Archive and Lifetime Surveillance of Astronaut Health (lsda.jsc.nasa.gov)
